# The Role of Metarepresentation in the Production and Resolution of Referring Expressions

**DOI:** 10.3389/fpsyg.2016.01111

**Published:** 2016-07-27

**Authors:** William S. Horton, Susan E. Brennan

**Affiliations:** ^1^Department of Psychology, Northwestern UniversityEvanston, IL, USA; ^2^Department of Psychology, Stony Brook UniversityStony Brook, NY, USA

**Keywords:** reference, common ground, metarepresentation, memory, cognitive models

## Abstract

In this paper we consider the potential role of metarepresentation—the representation of another representation, or as commonly considered within cognitive science, the mental representation of another individual's knowledge and beliefs—in mediating definite reference and common ground in conversation. Using dialogues from a referential communication study in which speakers conversed in succession with two different addressees, we highlight ways in which interlocutors work together to successfully refer to objects, and achieve shared conceptualizations. We briefly review accounts of how such shared conceptualizations could be represented in memory, from simple associations between label and referent, to “triple co-presence” representations that track interlocutors in an episode of referring, to more elaborate metarepresentations that invoke theory of mind, mutual knowledge, or a model of a conversational partner. We consider how some forms of metarepresentation, once created and activated, could account for definite reference in conversation by appealing to ordinary processes in memory. We conclude that any representations that capture information about others' perspectives are likely to be relatively simple and subject to the same kinds of constraints on attention and memory that influence other kinds of cognitive representations.

## Referring and representation

**Figure 1:**
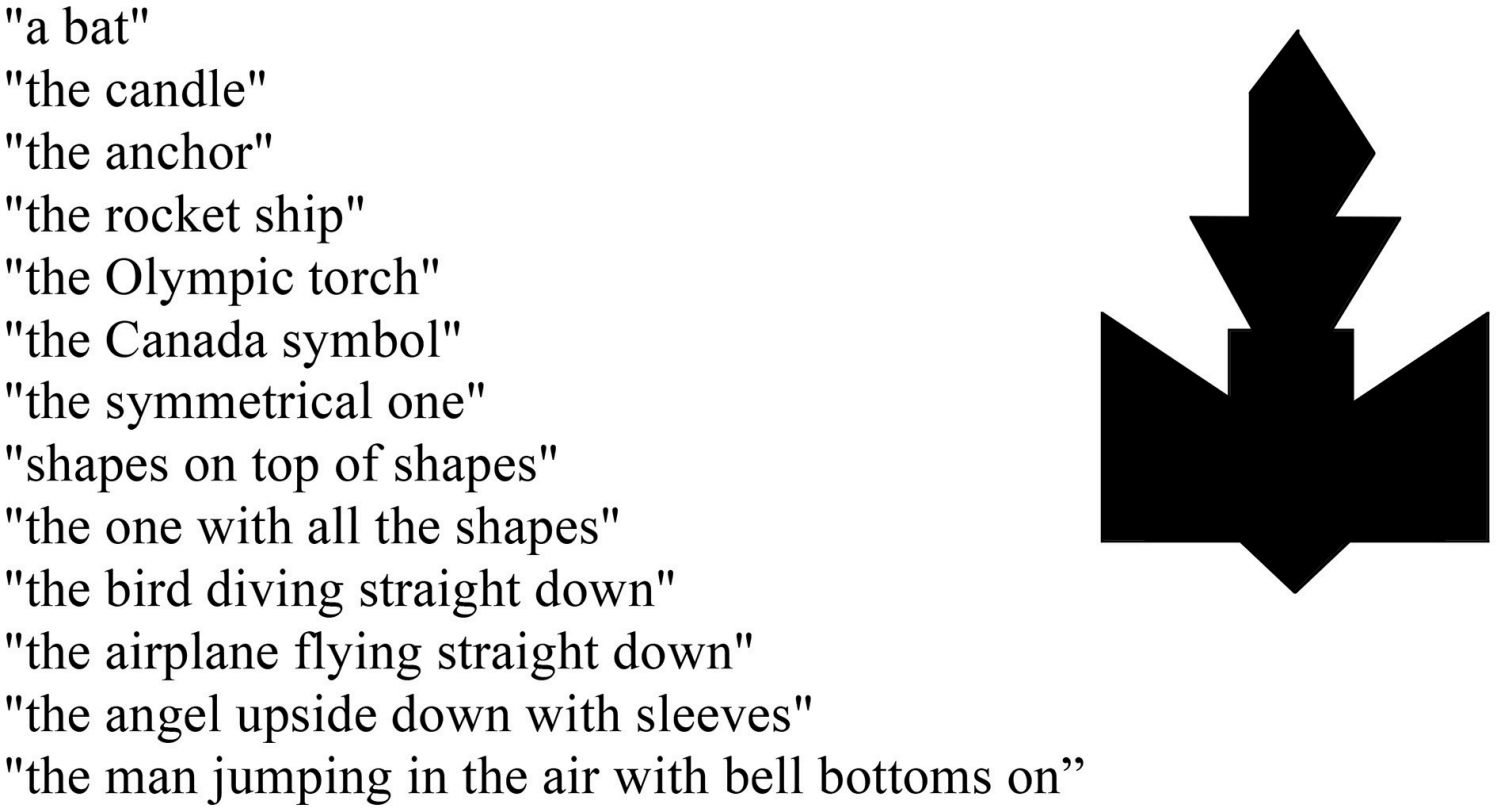
**Stellmann and Brennan (1993), unpublished data**.

Speakers have many options in the production of referring expressions, ranging from simple pronouns to complex definite or indefinite noun phrases. Moreover, there is potential for substantial variability in the noun phrases speakers choose. Consider just a few of the referring expressions for the novel object in Figure 1, each used by a different pair of speakers.

Successful referring—with the desired effect of getting a speaker and an addressee focused on the same referent—is as much about an interactive *process* as it is about a *product*. The expressions in Figure 1 emerged from dialogues such as the following, collected during an experiment by Stellmann and Brennan (1993), in which a director, A, is trying to help a matcher seated behind a screen, B, to match a dozen cards with abstract geometric figures (tangrams) into a target order (Note: Overlapping speech occurs within ^*^asterisks^*^).

A: Number 8…is a candle?
B: A candle…ok…
A: It's got a lot^*^of^*^
B:^*^is it^*^ wider on top and short on the bottom and it has, like, a diamond sticking out from the top?
A: Yes but rotate it the other way and so it's wider on the bottom
B: Wider on the bottom, hold on…
A: If you um for instance it has um
B: Which one is um number 8
A: This is number 8 if you want to think of it also it looks like someone doing a split on the ground or jumping in the air, yeah, with bell bottoms on
B: Which direction?
A: ^*^uh^*^
B: ^*^oh^*^ alright, alright, I see it, I see it
A: Ok
B: It looks like it's doing a split in the air
A: Right ^*^exactly^*^
B: ^*^alright^*^

Here, A begins by proposing that the object in question resembles a candle, but B is unable to recognize any suitable object in the set and asks for clarification. A, guessing which object B might be considering, suggests rotating the card. B attempts to discuss the card's geometric details, but after a few exchanges focused on the geometry of the object, A tosses out a new counter-proposal (*someone doing a split on the ground or jumping in the air, yeah, with bell bottoms on*). B eventually confirms this perspective and places the corresponding card on the board; this action provides evidence for the success of the referring process. In this exchange, after 16 conversational turns, speaker and addressee have finally come to believe that they mean the same thing. Thereafter, A and B referred to this object again on subsequent rounds as follows (note that the exchanges in these rounds are each separated by matches to an average of 11 other objects):

Round 2: B: The person with the bell bottoms doing a split in the air
                 A: Ok um wait alright ok
Round 3: A: The person with the bell bottoms jumping in the air
                 B: Got it
Round 4: B: The bell bottomed jumper
                 A: I had it there man

This example is representative of how 26 pairs of subjects in this experiment referred to the 12 objects that they repeatedly matched over four rounds. The processes through which people interactively seek and provide evidence for shared perspectives during referring in conversation is known as *grounding* (Clark and Wilkes-Gibbs, 1986; Clark and Schaefer, 1989; Clark and Brennan, 1991). The consistency among referring expressions produced by A and B across rounds, as well as the evidence provided in each round by the successful match of the matcher's object to the director's, suggest that for each object, the two partners built up common ground that enabled them to mentally represent the object in the same way (or highly similar ways). As the same time, the variability of expressions in Figure 1 suggests that other pairs conceptualized this object quite differently. Through such processes of conceptual coordination, partners converge on, and re-use the same terms within a conversation, displaying lexical and conceptual *entrainment* in their choices of referring expressions (Garrod and Anderson, 1987; Brennan and Clark, 1996).

What sorts of cognitive mechanisms support such coordination? There have been several types of answers to this question. Perhaps the simplest is one that appeals to direct cross-speaker activation of particular expressions, as proposed by the *interactive alignment* account (Pickering and Garrod, 2004). This account presumes that interlocutors converge on the same referring expressions simply because one speaker's utterances can automatically prime similar responses from the other, facilitating similar discourse representations over time (assuming that the interlocutors are similar). A compatible view, from Brown and Dell (1987; Dell and Brown, 1991), suggests that what appears to be partner-specific coordination is often actually *generic*, in that what is easy for a speaker to produce tends to be easy for an addressee to understand. Relevant claims for this view include that “speakers and listeners do not routinely take common ground into account during initial processing” (Pickering and Garrod, 2004, 179), and that “normal conversation does not routinely require modeling the interlocutor's mind” (ibid, p. 180). Pickering and Garrod's proposal is consistent with models that propose that common ground is used only *on demand*, when repair is needed (e.g., Brown and Dell, 1987; Horton and Keysar, 1996; Keysar et al., 2000):

“Establishment of full common ground is, we argue, a specialized and non-automatic process that is used primarily in times of difficulty (when radical misalignment becomes apparent)…speakers and listeners do not routinely take common ground into account during initial processing…full common ground is only used when simpler mechanisms are ineffective” (Pickering and Garrod, 2004, p. 179).

Much experimental work by Keysar, Barr and colleagues (e.g., Keysar et al., 2000; Barr and Keysar, 2002; Keysar et al., 2003) has been presented in support of this idea, suggesting that interlocutors in conversation behave egocentrically (at least at first), but that this does not hamper communication as long as interlocutors are similar enough and happen to inhabit the same context (see also Shintel and Keysar, 2009).

Another type of answer to the question of conversational coordination involves the notion of *metarepresentation*, which generally refers to the representation of another representation. Sperber (2000, p. 3) identified four main categories of metarepresentation: “Mental representations of mental representations (e.g., the thought, “John believes that it will rain”), mental representations of public representations (e.g., the thought, “John said that it will rain”), public representations of mental representations (e.g., the utterance, “John believes that it will rain”), and public representations of public representations (e.g., the utterance, “John said that it will rain”).” Accounts of language use often assume, either implicitly or explicitly, that definite reference requires some form of the first of these, or the mental representation of another's *mental* representation—typically considered as the representation of another person's knowledge, needs, or beliefs. Certainly, Grice's (1975) original notion of conversational implicature was inherently metarepresentational in this sense, being rooted in the idea that pragmatic meanings involve direct consideration of what is mutually known between rational speakers. Similarly, linguistic theories of reference production frequently assume that choices in the form and content of referring expressions emerge from speaker's assessments of the accessibility of particular referents in the minds of their addressees (e.g., Gundel et al., 1993; Grosz et al., 1995). And models of communication and intention recognition in computational linguistics and logic have often been focused on providing formalizations of representations of other agent's epistemic states (e.g., Cohen and Perrault, 1979; Ditmarsch et al., 2007). An important question for all of these approaches, of course, is how well they succeed in capturing the kinds of knowledge state inferences that human interlocutors are likely to make in real time during genuine interactions.

Theories appealing to the mental representation of other's mental representations often presume detailed consideration of the needs, knowledge, or beliefs of a social partner. For example, the influential notion of *theory of mind*, as first articulated by Premack and Woodruff (1978), and refined further by Dennett (1978) and Pylyshyn (1978), refers to an individual's mental capacity to reason about the mental states of others, an ability that appears to follow a distinct developmental trajectory into adulthood (Wellman et al., 2001; Apperly, 2011, 2013). An even more complex way to think about metarepresentation invokes the recursive modeling of mutual knowledge (*I know X; you know X; I know that you know X; you know that I know X; I know that you know that I know X*; and so on); however, it is widely acknowledged that this sort of recursive reasoning would be so resource-intensive as to be implausible [see the debates in Smith (1982) on this *mutual knowledge paradox*, and Clark and Marshall's (1978, 1981) proposed solution involving inferences about co-presence].

Given the apparent ease with which interlocutors plan and resolve referring expressions in conversation, it might seem prudent to prefer the simplest account that relies on “priming” of referent-label associations across interlocutors. However, experimental corpora such as Stellmann and Brennan (1993) raise some key questions about the nature of the representations underlying referential communication that cannot be explained by simple associations alone (see similar evidence presented by Brennan and Clark, 1996 and Horton and Gerrig, 2005b).

To that end, it is important to note that Stellmann and Brennan's experiment actually involved quartets of speakers. Two additional subjects, C and D, matched the same cards at the same time as A and B, but in a neighboring room. For the item shown in Figure 1, C and D entrained on a perspective that they ended up labeling as *the anchor*. Crucially, after both pairs matched the cards for Rounds 1–4, they were split up and re-paired such that A and D completed Rounds 5–8 together, as did B and C. Of interest was whether there would be any savings in the linguistic effort needed to match these now-familiar objects. Here is what ensued between A and her new partner, D, in Round 5 immediately after the partner switch:

A: ah the second one looks like maybe a person jumping in the air who is wearing bell bottoms…or it could be a candle
D: Jumping with bell bottoms…
A: yeah…oh well, you know, cause it has two triangles coming from the left and the right, but they're, um, it looks like a person jumping…he's not- it's definitely symmetric down the middle…
D: oh man…
A: There's- and it's a- um, a diamond, a triangle, a rectangle, and two, ah, two triangles going from the left and right, you know, you could, ah…
D: You can't make a picture out of it?
A: ah let's see, if you put it on its side it looks like an E
D: an E?
A: yeah, no, let's call it an anchor *that's cool*
D: ok ^*^the anchor one^*^ yeah ok
A: ok it looks like an anchor
D: ^*^yeah^*^
A: ^*^that's cool^*^

What is striking is that the director, A, did not simply pick up where had she left off with B, with the concise expression that had worked most recently (“the bell bottomed jumper”)—even though this would presumably have corresponded to the strongest trace in memory (according to Garrod and Anderson, 1987 *output-input coordination principle*, a precursor to the interactive alignment theory). Instead, she proposed an indefinite referring expression marked as tentative by hedging (“*ah the second one looks like maybe* a person jumping in the air who is wearing bell bottoms”), as if to display sensitivity to the fact (that is, mentally representing) that she as yet had no common ground with D. She also proffers an alternative expression, “or it could be a candle.” Such *re-conceptualizations* have been observed in other referential communication studies that involve switching from an old to a new conversational partner (e.g., Brennan and Clark, 1996; Horton and Gerrig, 2005b; Gorman et al., 2013). After D failed to accept either of the (re-conceptualized) perspectives that A had discussed with her old partner, the new partners ended up converging on the perspective that D happened to entrain on earlier with C in the other room. Meanwhile, in that other room, B and C struggled valiantly (over 27 turns) to arrive at what might best be described as a hybrid perspective (*bell-bottom anchor*), which they continued to use in their next 3 rounds together. Although all of these people were, by then, individually quite familiar with the object in Figure 1, they still had to expend significant effort to ground their references to this object with their new partners in Round 5, just as with their initial partners in Round 1.

In the last stage of Stellmann and Brennan's experiment, the original partners were reunited for four final rounds, 9–12: A joined up again with B again, and C with D, whereupon they matched the same cards again. At that point, A and B reverted immediately and efficiently to the perspective they had entrained on previously, using “the guy with the bell bottoms jumping,” “the bell bottom,” “the bell bottomed guy,” and “the bell bottomed man” respectively in Rounds 9–12. Likewise, C and D immediately returned to the unadorned definite expression “the anchor” as soon as they got back together in Round 9. That pairs often switched back smoothly in Round 9 to the relatively short expression they had entrained upon in rounds 1–4 suggests that their representations included more than just the association of a referent and a referring expression (or the perspective it indexes), but information about the communication partner as well.

In this article, we suggest that simple associations are not sufficient to account for these and similar patterns of conversational referring. As an alternative possibility, we examine the role of metarepresentations in communication, and whether representations of a partner's goals, informational needs, or knowledge must, by necessity, involve the kinds of time-consuming inferences most commonly associated with theory of mind. We consider whether metarepresentations are created, maintained, and used routinely during conversational episodes, or only strategically in response to special circumstances such as evidence of misunderstanding. At the same time, we will appeal to an explanation relying on ordinary memory processes such as resonance (Ratcliff, 1978), and avoid positing any sort of “special” memory representation or separate stage of processing to account for the apparent effects of common ground upon referring in conversation. We conclude that a simple (meta) representation about a conversational episode (once created and activated) could be rapidly re-instantiated into the current discourse context via simple cues.

## Metarepresentation in theory of mind

As stated previously, the notion of metarepresentation has been especially important within the literature on theory of mind (ToM), which refers to the capacity to reason about the mental states of others. A large body of research has sought to answer such questions as whether theory of mind is a uniquely human capacity, whether deficits in theory of mind can help explain particular social and communicative disorders such as autism and schizophrenia, and whether theory of mind abilities are mediated by unitary, specialized neural circuits in the brain (e.g., Call and Tomasello, 2008; Frith and Frith, 2012; Baron-Cohen et al., 2013).

As a rule, ToM is fundamentally important for making sense of the social world. To give a simple example, imagine you observe someone pick up a key and walk with it toward a closed door. Based on your capacity for mental state attribution (and your knowledge of keys and doors), you might reasonably infer that this person has the intention of unlocking the door. Some (e.g., Perner and Ruffman, 2005; Penn and Povinelli, 2007) have argued that expectations about another's behavior could be generated primarily on the basis of associative or statistical knowledge concerning the kinds of actions that involve, for example, keys and doors, without being mediated by a theory of mind inference, although others have argued that simple associations or rules cannot account for the range of contexts across development in which young children, in particular, come to show evidence for perspective-taking (Baillargeon et al., 2010). Because a person's (private) intentions are not directly observable, one can only use observations of behavior to make inferences about the mental states giving rise to those behaviors. Making such inferences is a form of “mindreading”—reasoning about another's internal mental state—and is the hallmark of how people engage their theory of mind (Apperly, 2011).

In principle, mindreading can involve a wide range of mental state attributions, including inferences about emotional states, perceptual access, and desires and goals. The empirical literature on ToM (and certainly the developmental literature) has commonly focused on situations that involve deliberative, reflective assessments of another person's knowledge, most often involving “false belief,” in which another's knowledge conflicts with reality (Baron-Cohen et al., 1985; Wellman et al., 2001). For example, the classic Sally-Ann task (Wimmer and Perner, 1983) asks children to reason about Sally's belief concerning the location of an object, which, unbeknownst to her, has been moved from its original location. To pass this task, a child must recognize that Sally possesses an incorrect belief about the object's location, be able to understand that the question is asking where she *would* (rather than *should*) look for it, and respond accordingly to the experimenter's question. Quite clearly, this requires access to metarepresentations of Sally's beliefs. One must not only represent what Sally believes, but must also be able to appreciate how Sally's beliefs differ from one's own (and from reality). The representational and computational challenges involved in such situations have been cited as one reason why children don't consistently pass classic false belief tests until after the age of four (e.g., Call and Tomasello, 1999; Bloom and German, 2000).

Much of the literature on theory of mind, though, suggests that adults, at least, have the ability to construct and access representations of another's knowledge, even if they don't always apply this ability in situations that require puzzling out what another person is likely to believe, or how they are likely to act (Keysar et al., 2003). It is not immediately evident, though, whether the types of deliberative mental state attribution required by experimental tasks exploring false belief and theory of mind are qualitatively similar to the kinds of spontaneous inferencing regarding common ground that would seem to take place during routine conversation. Moreover, the types of mental state attribution commonly presumed within the literature on theory of mind have often assumed a view of metarepresentations as discrete, neatly packaged, deterministic bits of information about other people's knowledge and beliefs. Experiments exploring this capacity most often probe for “on-demand” inferences based on the presence or absence of knowledge, as in the classic Sally-Ann task, rather than measuring probabalistic inferences that occur spontaneously. Exceptions include work by Samson, Apperly and colleagues (e.g., Samson et al., 2010; Surtees and Apperly, 2012; see also van der Wel et al., 2014) who have shown that both children and adults can use extremely simple cues, such as the direction of attention, to rapidly generate inferences about mental states. Moreover, responding correctly to the classic Sally-Ann task requires the ability to respond to a question that depends on understanding modal verbs. When 3-year-olds are simply asked to act out “what happens next?” upon Sally's return to the hidden-object situation, they are more likely to demonstrate a spontaneous ability to reason about ToM and respond correctly (Rubio-Fernández and Geurts, 2013).

Such evidence suggests that, while it can take time to reason (on demand) about another person's knowledge or beliefs, once a relevant metarepresentation has been evoked, taking account of a partner context need not involve a time-consuming (or even a very mature) process of reasoning. This information can be used just like any other information in memory.

## Reference diaries and triple co-presence

Metarepresentation in some form is often invoked by accounts of referential communication. Perhaps the most influential account of definite reference comes from Clark and Marshall (1978, 1981), who observed, “people's memory must be organized to enable them to get access to evidence they will need to make felicitous references. To make or interpret definite references people have to assess certain “shared” knowledge. This knowledge, it turns out, is defined by an infinite number of conditions. How then can people assess this knowledge in a finite amount of time?” (Clark and Marshall, 1981, pp. 56–57). After rejecting recursively-achieved mutual knowledge as cognitively implausible, Clark and Marshall proposed that interlocutors take advantage of representations they called “reference diaries” that encode evidence for *triple co-presence*—or evidence that the speaker, addressee, and referent were “openly co-present together” (1981, p. 32). On this account, definite expressions (such as those in Figure 1) are constructed and interpreted against the common ground established by interlocutors through a heuristic that shortcuts the problem of computing mutual knowledge recursively. This heuristic is based on an inference that the parties in a conversation perceive or recall common ground based on what they've discussed together (linguistic co-presence), their experiences together in the same environment (physical co-presence), or their presumed socio-cultural overlap (community co-membership). *Prior* co-presence is established through previous experience together, whereas *potential* co-presence is evoked by a speaker's rational expectation that an addressee can use the current context to understand, for example, the intended referent of *I'd like that loaf of bread please* when accompanied by a pointing gesture over the addressee's shoulder (Clark and Marshall, 1981; for discussion, see Polichak and Gerrig, 1998). An inference on the part of a speaker that she and her addressee are contextually co-present presumably supports some kind of suitable representation of partner-specific information that facilitates *audience design* (Clark and Murphy, 1982; Bell, 1984; Horton and Gerrig, 2002), allowing her to produce referring expressions that a particular addressee is likely to be able to resolve. Such representations also allow addressees to interpret the same referring expression differently when it is spoken by different speakers in different contexts (Metzing and Brennan, 2003).

Clark and Marshall (1981) were not specific about the characteristics or limitations of possible partner-specific representations (which we call metarepresentations), apart from proposing that they encode triple co-presence (an association linking the self, an other, and the information in question). However, in their sections on *organization of memory* and *components of memory*, they referred to episodic memories of events that speakers and addressees have experienced together as “compartmentalized into useful units” that can be selectively accessed (p. 55), and that shift when the interlocutor changes in a conversation.

Ongoing debates in psycholinguistics have focused on the extent to which consideration of common ground *routinely* and *initially* informs language processing (Brennan and Hanna, 2009), or whether it is invoked in a separate stage of processing, like a repair (e.g., Brown and Dell, 1987; Keysar et al., 2000; Pickering and Garrod, 2004) Where Clark and Marshall and their critics agree is that sometimes effort must be expended in order to establish common ground or to propose or resolve a referring expression, but that frequently, referring in conversation seems effortless. The question remains as to what sort of representation underlies processing in this latter situation.

## A role for metarepresentations in referring

In principle, the notion of metarepresentation would appear to be central to models of reference and language use. An important question is whether considering metarepresentations need always be resource-intensive and time-consuming, or whether people can rapidly, and potentially automatically, behave as if they have access to appropriate metarepresentations under the right circumstances. In this section and the next, we argue that the metarepresentations themselves need not be elaborate or encode inferences about complex mental states, but can be simple and partial, driven by current conversational purposes. Furthermore, we propose that once a suitable episodic metarepresentation has been activated, it may be used as fluidly and rapidly as any other information available in memory.

In contrast, some have argued that speakers and addressees are inevitably “egocentric,” and that taking account of a partner's perspective as distinct from one's own can happen only as a kind of delayed processing or repair (Keysar et al., 1998; Epley et al., 2004). Shintel and Keysar (2009) point out that “elaborate reasoning that requires interlocutors to keep updated metarepresentations of the other's beliefs that are separate from their own representations of the situation is both time consuming and cognitively demanding.” Not surprisingly, experiments that place speakers in perceptual contexts with both salient privileged information *and* information that is in common ground with an addressee, along with the need to continuously distinguish these, show evidence for interference between dueling perspectives (Horton and Keysar, 1996; Keysar et al., 2000). Indeed, some have used this kind of evidence to argue for modularity in cognitive architecture (Barr, 2008).

A related argument is that what appears to be partner-specific processing (resulting, e.g., in entrainment and audience design) occurs simply when speakers and hearers happen to share the same context, as suggested by Pickering and Garrod's (2004) interactive alignment model (see also Brown and Dell 1987). As Pickering and Garrod argue, simple priming can facilitate convergence in referring expressions without obligating interlocutors to directly represent the other person's perspective. On these accounts, metarepresentations would have little role to play in fundamental aspects of language processing, instead being relevant only in the context of slower, more effortful processes of monitoring and repair.

In this context, we make two critical points about referring in conversation. The first critical point is that referring is not a deterministic process, but a collaborative one that involves coordination between (at least) two people. As a result, any form of metarepresentation that emerges from conversational grounding is likely to be highly probabilistic, in the sense that the relevant memory traces will vary in strength and accessibility. To understand why, we return to our examples from Stellmann and Brennan's corpus and focus more closely on what happened after the partner-switch at Round 5. Here, D began with a couple of proposals to her new partner, B; she first proposed the perspective that had worked well earlier with her former partner C, and then added another perspective that C had failed to take up:

D: ah the second one looks like maybe a person jumping in the air who is wearing bell bottoms…or it could be a candle
B: Jumping with bell bottoms…

Here, by echoing some of D's words hesitantly, B provides evidence of uncertainty; he's considering this proposal but isn't able to accept it. D responds by trying to motivate *the jumping man with bell bottoms* using a lengthy and laborious geometric description, but then B suggests returning to a figurative strategy:

B: You can't make a picture out of it?

After two more proposals, D fortuitously hits upon the perspective that B happened to use earlier with former partner A: “let's call it an anchor.” This works, and on they go. Examples like this underscore a critical aspect of Clark and Marshall's original co-presence account (Clark and Brennan, 1991; Clark, 1996), which is that heuristic-based approximations of what others know may suffice much of the time, given that grounding and the potential for interaction provide relatively inexpensive ways to recover *if* and *when* interlocutors get it wrong. Speakers' current purposes often don't require them to be perfect or to work too hard on the inferences they make. They are thus able to balance the costs of delaying an utterance (in order to plan it more fully) with the risks of appearing to be inattentive or losing their partners' attention or running out of time in a task (Clark and Brennan, 1991).

A second critical point is that evidence for representation can be found in *how* a speaker presents a particular referring expression. Referring expressions can be fluent, disfluent, brief, wordy, hedged, or presented with falling or question intonation (Smith and Clark, 1993; Brennan and Williams, 1995). As the examples from Stellmann and Brennan's corpus illustrate, a referring expression, once proffered, has the potential to be (and be marked as) tentative, vague, or unacceptable, suggesting that any representation of another's knowledge that might emerge through conversational grounding also has the potential to be incomplete or incorrect. Moreover, given that referring expressions and other types of utterances are generally produced and understood on a time scale that would seem to preclude lengthy deliberation, there must be processes that permit reasonably rapid access to perspective-relevant information. These factors impose critical constraints upon any account attempting to capture the nature of successful referring in language use.

As the speakers in Stellmann and Brennan's study transitioned from one partner to another and back again, the way in which they framed referring expressions changed accordingly, as described previously. One possible explanation for the hedging after the first partner change in Round 5 is that the presence of the brand-new partner, D, weakened the memory trace for the previous referring expression. However this explanation by itself is not so convincing, as the repeated referring in Rounds 1–4 with partner B should have rendered A's memory for what to call this object quite strong. Moreover, the return to Partner B in Round 9 (when the original pairs were reunited) showed little to no disruption due to the partner switch, but rather a smooth return to the originally entrained-upon perspective. Reverting to this original perspective might be expected to fight against one's memory traces for the repeated (and more recent) references to *the anchor* in Rounds 5–8. However, a plausible explanation is that the partner-switch successfully boosted accessibility of the previous episode, likely through a *compound cue* comprised of the current referent plus the presence of the original partner B (Ratcliff and McKoon, 1988; Horton and Gerrig, 2005a, in press).

At issue is when and how such a shift in referring expressions also might be shaped by remembering that the original partner shares a particular perspective on the tangrams not shared by the other partner. If so, we can ask if and when this belief is encoded as part of the original memory trace for this interaction. The tentative way in which A expresses “looks like maybe a person jumping in the air who is wearing bell bottoms” is presumably *not* due to A's own lack of facility with this perspective (which, after all, A had just finished deploying over and over with B), but may, in fact, be caused by A's sense that a new partner, D, would not find it so easy to take this perspective (in other words, that D is not implicated in A's episodic representation). In principle, the difference between proposing the efficiently packaged definite expression “bell bottomed jumper” vs. “looks like maybe a person jumping in the air who is wearing bell bottoms” would seem to involve ready access to suitable representations that encode such beliefs about another's knowledge. As we suggest, though, the availability of such representations is likely to be influenced by the nature of the grounding processes that give rise to these beliefs, providing speakers with the opportunity to encode inferences concerning what can be taken as shared—especially when these inferences are supported by salient features of the conversational context.

It is not clear how this apparently partner-specific effect on the forms of utterances would be handled by Pickering and Garrod's simple priming account, or by the “output-input coordination” principle of Garrod and Anderson (1987), which predicts that a speaker should continue to use the same expression that worked last time (regardless of addressee). As we argue in the next section, though, there are good reasons to eschew an account of common ground processing that relies on *detailed* metarepresentations of other's knowledge. But, assuming much simpler types of representations for purposes of conversational interaction need not doom individuals to egocentrism. Both of the accounts that we describe next support ways in which “ordinary” partner-relevant representations could give rise to felicitous language use that are consistent with constraints based on cognitive capacities of individual speakers and salient features of conversational contexts.

## Ordinary memory and “one bit” representations

The examples from Stellmann and Brennan's corpus demonstrate that when speakers use a referring expression, they can depend on their addressees to let them know if a referent is unclear. They can take the risk of starting to speak in a timely manner, designing referring expressions based on available representations that are likely (but not guaranteed) to work. If we accept that ordinary conversational reference is unlikely to occur in a resource-intensive manner qualitatively similar to the deliberative consideration of false belief, and if we accept that low-level priming explanations such as output-input coordination (Garrod and Anderson, 1987) or interactive alignment (Pickering and Garrod, 2004) fail to adequately account for audience design, the question remains as to how interlocutors so often are able to refer to objects in ways that are generally consistent with shared knowledge. Here, we consider two accounts that do not see fully elaborated partner models as being necessary for particular referring expressions to succeed—indeed, both emphasize the fact that, in conversation, success is not guaranteed. Specifically, these accounts are the “memory-based” account described by Horton and Gerrig (2005a, in press), and the “one-bit” account described by Galati and Brennan (2006; 2010; see also Brennan and Hanna, 2009).

These two accounts complement each other, in that both connect the dots between memory representations and audience design, with each emphasizing a different launching point: The memory-based account begins with ordinary memory processes and representations, in order to consider how apparent instances of audience design emerge fluidly in conversation, whereas the one-bit account begins with audience design or partner specific processing in conversation, in order to consider what sorts of context-augmented representations (or common ground) could underlie ordinary language processing. Common to both accounts is the idea that partner-specific referring in conversation is mediated through ordinary memory representations (without appeal to special or elaborate mechanisms of the kind entailed by the notion of reference diaries or theory of mind). Both accounts reject as implausible (and computationally expensive) the idea that people routinely “tag” (and continually make triple co-presence inferences about) every element of information that could be relevant to “common ground.” Both accounts endorse the view that relevant representations frequently include contextual information concerning a conversational partner, but that there is nothing qualitatively “special” about this information that gives it priority over other relevant information (see Goldinger, 1998 for a similar view).

Moreover, the extent to which person-centered information might be individuated in ways that reliably support felicitous reference will, on both accounts, depend greatly on factors such as immediacy, relevance, perceptual vividness, and goals, as well as whether such information has already been processed in the conversational context. In these respects, both the one-bit view and the memory-based view highlight how routine cognitive considerations strongly shape the extent to which people show evidence for consideration of common ground. On these accounts, successful definite reference depends on simpler representations that support rapid access to contextually relevant knowledge.

*The memory-based model* (Horton and Gerrig, 2005a, in press) is a cognitively motivated account explaining how language users could gain access to partner-relevant information in ways that require neither special-purpose representations nor special-purpose processes. More specifically, Horton and Gerrig (2005a) described how considerations of common ground could occur on the basis of ordinary representations as they become accessible from memory through ordinary means. For instances of “personal” common ground in particular (Clark, 1996), these memory representations were characterized as rich episodic traces of previous encounters with others, representing the products of the kinds of encoding typical of one's experiences of particular events.

Once such traces are encoded into memory, subsequent encounters can trigger the automatic retrieval of relevant memory traces, through a process termed *resonance*. Inspired by cue-driven retrieval processes found in models of recognition memory (Ratcliff, 1978; Gillund and Shiffrin, 1984; Hintzman, 1986; Ratcliff and McKoon, 1988), resonance involves the parallel activation of information that shares overlapping features with the memory probe (e.g., the presence of an interlocutor). When resonance reaches some threshold, which itself is a function of the recency and frequency with which a memory has been previously retrieved, this information can become accessible in a way that influences other processes. On this account, then, implicit “assessments” of common ground emerge from a speaker's automatic sense that particular information can be treated as familiar or not within a particular context. This in turn can lead speakers to use particular forms of reference if relevant linguistic representations become sufficiently accessible via resonance within a time course that can affect planning and production. Critically, though, under this proposal audience design does not require that these representations be specifically tagged with respect to common ground, although relevant memory traces may still happen to encode partner-relevant information.

*The one-bit proposal* (Galati and Brennan, 2006, 2010; Brennan and Hanna, 2009) arose from the observation that, in experiments designed to distinguish the perspectives of conversational partners, common ground seems to be able to rapidly guide comprehension and planning of referring expressions when conditions differ by one or just a few well-established, relevant cues that in these experiments happened to be binary—for example, *my partner is a native speaker of English*, or not (Bortfeld and Brennan, 1997); *my partner can see what I'm doing*, or not (Brennan, 2005); *my partner and I have the same spatial perspective*, or not (Schober, 1993; Duran et al., 2011); *my partner can reach the object she's talking about*, or not (Hanna and Tanenhaus, 2004); *my partner can see a picture of what we're discussing*, or not (Lockridge and Brennan, 2002); *I have talked about this with my partner before*, or not (Horton and Gerrig, 2002; Metzing and Brennan, 2003; Galati and Brennan, 2006; Matthews et al., 2010); or *my partner and I were interrupted before we finished discussing this*, or not (Brown-Schmidt, 2009). Such contextual cues, especially if they're established as relevant through perceptual salience or having made a previous inference, can support the rapid access and use of episodic information in order for speakers to design an utterance *for* a particular audience (as opposed to behaving “egocentrically”), or in order for addressees to adapt the processing of a referring expression with a particular speaker in mind.

But even for the simplest of metarepresentations (e.g., *she can/cannot see me talking*), in order for such partner-specific information to guide processing, it must already be accessible (Horton and Gerrig, 2002); this means that the first time an inference is needed, this is likely to require additional processing time. This was demonstrated in a referential communication study (Hwang et al., 2015) in which Koreans who spoke English as a second language worked with a native English speaker to match labels that would be unpronounceable in Korean (which lacks not only any coda-final /b/ vs. /p/ contrast, but also any contrast between the vowels /æ/ and /ε/). The Korean speakers did not spontaneously produce recognizable contrasts unless they had just been primed with a similar sound by the native-English-speaking partner, or *unless there was a pragmatic reason to do so*, in order to make a relevant pragmatic distinction that their partners needed to do the matching task—for example, to distinguish a card labeled *bib* from a nearby *bip*. Critically, the first time they encountered their partner's pragmatic need, it took them significantly longer to initiate speaking; but when a similar pragmatic contrast (between different items) was needed after that, they were just as fast to initiate speaking as with other, baseline expressions. This evidence supports the idea that a representation of the discourse context that includes pragmatic information that has already been perceived or computed can rapidly shape referring without the need for elaborate, computationally expensive inferences (see Brennan and Hanna, 2009; Shintel and Keysar, 2009).

Thus, the one-bit account presumes a role for metarepresentations of episodes relevant to conversational contexts, permitting language use to be shaped by inferences that concern what other people might know. In particular, these inferences are most likely to occur either *before* a speaker formulates a referring expression (or an addressee interprets it), based on salient percepts about their physical co-presence, or at the time when a speaker or an addressee is first prompted to consider pragmatically-relevant differences, especially differences that are relatively simple and supported by a stable conversational context. At the same time, however, any metarepresentations that encode such inferences are subject to the constraints of ordinary memory; that is, they are likely to vary in strength and be schematically focused on critical features of the interactive setting. Once evoked, they may support the rapid or automatic use of partner-specific information in similar contexts [as suggested by the Hwang et al. (2015) results described above], rather than requiring additional laborious inference. For example, if an inference about common ground has already been made, or if relevant evidence is perceptually salient, then such information could be available to be used in the formulation and interpretation of referring expressions without further delay (Galati and Brennan, 2006; Brennan and Hanna, 2009). This can result in rapid and “smart” (rather than slow and laborious) adaptations of utterances to a particular partner's needs, perspective, or context.

Critically, the rapid use of partner-specific knowledge under these circumstances could readily occur via the kinds of memory-based processes described by Horton and Gerrig (2005a). Even if metarepresentations are not *always* deployed in language use, the number and variety of findings showing context-appropriate uses of perspective (see discussion in Brennan and Hanna, 2009; Brown-Schmidt and Hanna, 2011) demand that researchers provide a psychologically-plausible account of when and how speakers might come to consider inferences about others' knowledge. For its part, Horton and Gerrig's (2005a) memory-based account does not deny the possibility of metarepresentation. Indeed, in their description of *strategic* assessments of a partner's knowledge, they identified several instances in a corpus of telephone conversations in which speakers appeared to consult (or, more likely, construct) representations of what their addressees might know (e.g., “Yeah, I've got another buddy who, uh, is a Marine pilot. I'm trying to think if you had ever met this guy”). The suggestion, however, was that such activity was likely to be too computationally effortful and/or costly to provide a general account of audience design.

Even so, the primary focus of the memory-based view concerned the automatic accessibility of partner-relevant information via resonance. Much of the time, these partner-relevant representations will be limited to episodic traces of previous encounters with others, allowing individuals to show evidence for partner-sensitivity without requiring detailed inferences about common ground. In principle, though, because resonance is a “dumb” process that works on whatever information is available in memory, there is nothing inherent about resonance as a process that would prevent it from facilitating the retrieval of representations that capture inferences about the knowledge of certain partners—as long as such information is part of the memory trace.

One of the fundamental observations about resonance as an ordinary memory process is that the types of information that become accessible via resonance are likely to be highly dependent upon features of the conversational context, both in terms of encoding strength as well as the availability of appropriate retrieval cues. Thus, certain conversational situations might not only be more likely to result in stronger memory traces for particular sources of information (Horton and Gerrig, 2005b), but might also unfold in a way that supports the direct encoding of highly constrained inferences concerning other's beliefs about that information. In particular, aspects of conversational grounding described by Clark and colleagues could under many circumstances provide the right setting for particular metarepresentational inferences to be encoded as part of the episodic trace for particular interactions (as first suggested by Clark and Marshall, 1981). For example, explicit indications that an individual has understood a particular conceptual perspective (e.g., “alright I see it, I see it” in the opening example) might be more likely to lead to the encoding of the belief that this speaker views that referent in this way. It would be important to empirically distinguish this, though, from the simpler possibility that particular kinds of feedback may just generally lead to stronger memory traces for the interaction.

One piece of evidence on this point comes from Brown-Schmidt (2012), who showed that participants generated stronger inferences about shared knowledge in situations in which they responded to direct questions from a confederate speaker, consistent with the idea that common ground is mediated via “gradient” representations. Thus, while information about simple verbal events such as “Sally called this *an anchor*” would, on any account, almost certainly be present in the episodic trace, processes of negotiating reference might enable information such as “Sally believes this can be conceptualized as an anchor” to (probabilistically) become part of the trace as well. The probabilistic nature of common ground is an underappreciated part of Clark and colleague's original theory, which models the strength of evidence interlocutors provide about their understanding and uptake during conversational interaction (Clark and Schaefer, 1989; Schober and Clark, 1989; Clark and Brennan, 1991; Brennan and Clark, 1996). As meanings are grounded, speakers provide metalinguistic cues as to their commitment to the content of their utterances (Smith and Clark, 1993), and hearers accurately understand and use such cues (Brennan and Williams, 1995; Swerts and Krahmer, 2005). So even though cognitive restrictions prevent individuals from encoding anything that resembles the infinite regress of mutual knowledge, they may be able to use metalinguistic cues and co-presence heuristics to estimate mutual knowledge.

Such cognitive restrictions tie in directly to the motivation for the one-bit account as proposed by Galati and Brennan (2006, 2010). Clearly, ordinary memory representations of particular social interactions cannot encode every possible inference concerning other people and potential referents as part of a simple metarepresentation. But if the conversational context supports a particular inference concerning the likely knowledge of others as relevant, then it is possible that language users may easily encode such inferences as part of the partner-specific memory trace and use it automatically in reference resolution. That is, once so-called metarepresentational information has been computed, it can be available for subsequent retrieval via ordinary resonance, as described by the memory-based model. As such, this retrieval need not be guided in the moment by explicit deliberations about another's perspective.

We wish to stress, though, that the types of representations of another's knowledge likely to be most relevant for everyday language use will most generally be quite different from the sorts of discrete, all-or-nothing inferences about mental states commonly presumed in the literature on theory of mind. That is, we believe that variation across conversational contexts, as well as within conversations over time, will shape the kinds of partner-specific information that become accessible for particular speakers as they formulate and comprehend utterances. Under these circumstances, some aspects of metarepresentational beliefs will be more immediately accessible than others, and any such knowledge is likely to be partial or schematic, depending on the nature of memory cues present in the conversational situation as well as the strengths of the underlying traces stored in memory. With repeated referring, memory traces are stronger, and so any “conceptual pact” achieved by two conversational partners to refer to a particular referent with a particular label is likely to be stronger and more easily evoked (Brennan and Clark, 1996), consistent with the probabilistic or gradient nature of common ground (Brown-Schmidt, 2012). Moreover, what might be seen as computationally expensive (such as keeping track of individual information) need not be so, if ordinary memory processes also support the binding of relevant contextual factors as part of the same representation. But, with highly similar episodes involving context switches (such as interactions with different partners), distinctions between relevant representations might become blurred, leading to further opportunities for interference leading to source-monitoring errors or egocentric mistakes.

Our converging viewpoints emphasize the fact that consideration of another's knowledge is rarely likely to be a discrete, all-or-nothing event, instead unfolding over time as cues become available in the conversational context, leading to the retrieval of partner-focused representations that are incremental and dynamically changing as new information comes online. Furthermore, we suggest that many of these inferences are likely to be simple, reusable, and highly supported by salient features of the conversational context. Once these inferences have been computed and encoded as part of the memory trace for that interaction, the resulting metarepresentations, whether schematized or not, are potentially available for retrieval via ordinary memory-based processes. As a function of ordinary memory, this retrieval will be highly dependent upon the presence of appropriate cues and can still wax and wane as the conversation proceeds.

## Conclusions

By taking seriously questions about the nature of representations to which language users have access for purposes of conversational reference, our aim has been to emphasize the extent to which such representations are constrained in a number of ways by ordinary memory, consistent with constraint-based approaches to reference and common ground (Hanna et al., 2003; Brown-Schmidt and Hanna, 2011). Such constraints are not only internal, tied to fundamental cognitive processes of attention and memory, but also external, arising from the conversational situation (including processes of grounding meanings with a partner). In particular, the issue of metarepresentation highlights key questions about the nature of the ordinary memory traces that potentially encode inferences about another's knowledge. Another person's knowledge or perspective may well have the same status in a metarepresentation as any other relevant aspect of the context in which referring takes place, or it may be supported by distinct neural circuitry (for discussion, see Brennan et al., 2010). Neuroscience has begun to explore the neurocognitive markers associated with particular types of socially relevant capacities, including theory of mind (e.g., Ruby and Decety, 2003; Baron-Cohen et al., 2013; Hamilton et al., 2014). It remains to be seen whether representations of (or inferences about) other people's knowledge, needs, or beliefs are qualitatively different from any other contextual representations or inferences required by language use in conversation.

## Author contributions

All authors listed, have made substantial, direct and intellectual contribution to the work, and approved it for publication.

## Author notes

This material is based on work done while SB was serving at the National Science Foundation. Any opinion, findings, and conclusions or recommendations expressed in this material are those of the authors and do not necessarily reflect the views of the National Science Foundation. We thank Richard Gerrig and the two reviewers for their comments on an earlier draft.

### Conflict of interest statement

The authors declare that the research was conducted in the absence of any commercial or financial relationships that could be construed as a potential conflict of interest.
